# The Effect of Transcranial Direct Current Stimulation on Lower-Limb Endurance Performance: A Systematic Review

**DOI:** 10.3390/bioengineering11111088

**Published:** 2024-10-30

**Authors:** Zhen Xu, Bin Shen, Songlin Xiao, Chuyi Zhang, Jianglong Zhan, Jingjing Li, Weijie Fu, Jing Jin

**Affiliations:** 1 School of Exercise and Health, Shanghai University of Sport, Shanghai 200438, China; xuzhen0226@163.com (Z.X.); shenbin614@163.com (B.S.);; 2 Key Laboratory of Exercise and Health Sciences of Ministry of Education, Shanghai University of Sport, Shanghai 200438, China; 3 School of Psychology, Shanghai University of Sport, Shanghai 200438, China

**Keywords:** transcranial direct current stimulation (tDCS), lower limbs, endurance performance, knee joint

## Abstract

This study systematically reviews the literature on transcranial direct current stimulation (tDCS) interventions for lower-limb endurance performance in healthy adults and provides a summary of the effects and underlying mechanisms of tDCS on lower-limb endurance performance. Systematic searches were performed in PubMed, Web of Science, EBSCO, and ScienceDirect. The risk of bias was assessed using the Cochrane risk of bias assessment tool. The electronic search totaled 341 studies. Twenty-one studies were included in the review after screening. The results show that tDCS effectively improved time to task failure (TTF), increased blood lactate accumulation, and reduced the rating of perceived exertion during cycling. However, the tDCS failed to significantly improve the TTF, relieve muscle pain, and reduce fatigue indices during single-joint fatigue tasks in the knee. Moreover, tDCS intervention caused the effective improvement of the overall lower-limb endurance performance but exerted no uniformly conclusive effect on knee endurance performance. This finding can be partly attributed to varying stimulation protocols across studies. Future studies may focus on the effects of the application of stimulation protocols, such as multitarget stimulation and personalized dosage, to develop targeted stimulation protocols.

## 1. Introduction

Fatigue is one of the most important factors affecting sports performance [1]. Exercise fatigue of the lower limb leads to changes in its mechanical characteristics [2,3], which is accompanied by a decrease in balance control capability and proprioceptive function; these modifications seriously restrict healthy adults, especially athletes, from obtaining satisfactory sports performance and competitive results [4,5]. Therefore, mechanisms for improving lower-limb fatigue resistance during endurance exercise have become an important concern in the field of human sports biomechanics.

Transcranial electrical stimulation, encompassing transcranial direct current stimulation (tDCS) [6], alternating current stimulation [7], and random noise stimulation [8], is a non-invasive neuromodulation technique that can induce hyperpolarization or depolarization of the neuronal resting membrane potential, depending on the polarity of the stimulating electrodes [9]. This technique is widely used across various fields due to its safety, cost-effectiveness, and ease of control of stimulation parameters [10]. In recent years, tDCS has been introduced as a neuro-biomechanical enhancement technique for improving human capabilities [11,12]. It can increase the excitability of the primary motor cortex, improve balance performance, and accelerate motor learning [13,14]. In addition, tDCS increases M1 excitability, which reduces the reliance on the supplementary motor area (SMA) and decreases an individual’s perception of effort during task execution [15].

Most current studies have investigated the influence of a tDCS session on endurance performance via a single-joint laboratory exercise test of the upper or lower limbs. tDCS considerably prolongs the time to task failure (TTF) in endurance exercises [16,17,18,19]. However, some studies discovered inconsistent conclusions, which suggests that tDCS fails to extend the TTF in endurance exercises [20,21]. A few works have explored the neurophysiological mechanisms underlying the action of tDCS in combination with brain imaging techniques. A study based on functional near-infrared spectroscopy found that tDCS improves the efficiency of neuronal transmission in bilateral sensorimotor cortex [22]. tDCS enhances endurance performance through the increase in corticospinal excitability [19,23,24]. However, other studies reported the lack of a notable association between the improvement of endurance performance and corticospinal excitability [16]. Results regarding the effects of tDCS on endurance performance also show inconsistency, and the potential mechanisms remain unclear. Existing reviews focused on summarizing the findings of tDCS on comprehensive motor abilities (e.g., muscle strength, endurance, explosive power, etc.). Systematic summaries and the exploration of potential mechanisms for tDCS interventions that specifically target the important abilities of the lower limbs (e.g., endurance) are lacking [25,26].

This study systematically reviews the literature on tDCS interventions for lower-limb endurance performance in healthy adults and summarizes the effects of tDCS on both overall lower-limb endurance and single-joint endurance (knee and ankle). This systematic review serves as a reference for the exploration of the potential mechanisms by which tDCS affects lower-limb endurance performance and the design of future studies.

## 2. Methods

### 2.1. Search Strategy

The systematic review protocol and reporting adhered to the Cochrane Handbook for Systematic Reviews of Intervention and Preferred Reporting Items for Systematic Reviews and Meta-Analyses guidelines [27]. The literature search was conducted in the following electronic databases: PubMed, Web of Science, EBSCO, and ScienceDirect, up to April 2024. The search was conducted using the terms (“transcranial direct current stimulation” OR “tDCS”) AND (“endurance” OR “fatigue”) AND (“leg” OR “lower limb” OR “knee” OR “ankle” OR “foot”) in all databases.

### 2.2. Eligibility Criteria and Article Selection

Studies were selected in accordance with the following inclusion criteria based on study design, participants, intervention, comparison, and outcome measures: (1) randomized control trials; (2) healthy adults as participants; (3) the use of tDCS intervention and comparison with sham tDCS (i.e., placebo); and (4) indicator of lower-limb muscular endurance performance as the primary outcome. Exclusions comprised the following: conference abstracts, meta-analyses, reviews, letters, case reports, animal studies, or non-English publications.

Two researchers (ZX and BS) independently screened the titles and abstracts of studies determined through the search strategies after the removal of duplicates. If an abstract met the inclusion criteria, then the full text of the article was reviewed for confirmation. The authors resorted to discussion with a third researcher (WF) to resolve conflicts and disagreements, and all authors agreed on the final included studies.

### 2.3. Data Extraction

A summary of raw data from the included articles was prepared, and the results were divided into two categories: (1) the effect of tDCS on the endurance performance of the overall lower limbs and (2) the influence on single joints/segments of the lower limbs. This categorization facilitated interpretation of the findings. In addition, the following data were extracted: first author, publication year, sample size, gender, age range, tDCS protocol (e.g., electrode position, size, current intensity, duration), and primary outcome measures.

### 2.4. Risk of Bias Assessment

The Cochrane risk of bias assessment tool, which includes the following aspects, was used to assess the risk of bias for each study: random sequence generation, allocation concealment, blinding of participants and personnel, blinding of outcome assessment, incomplete outcome data, selective reporting, and other biases. Each aspect was evaluated as high risk (+), low risk (−), or unclear risk (?) of bias using Review Manager 5.4 software.

The risk of bias was assessed independently by two researchers (ZX and BS). In cases of disagreement, a third experienced researcher (WF) conducted their own assessment to reach a consensus.

## 3. Results

**Figure 1:**
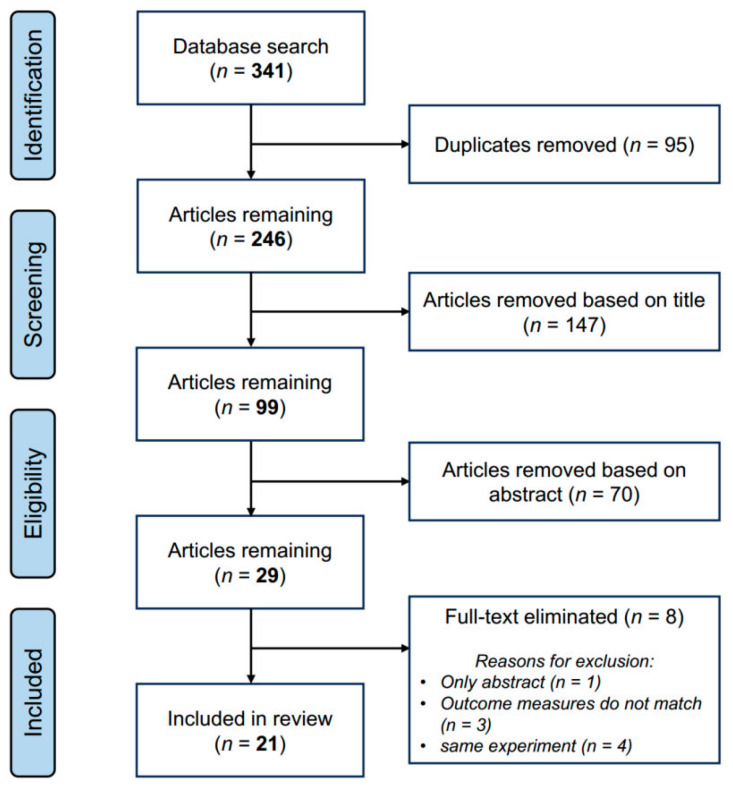
Flow diagram of the search strategy.

The database search yielded 341 related articles (26 in PubMed, 47 in Web of Science, 86 in EBSCO, and 182 in ScienceDirect). A total of 21 articles [18,19,20,28,29,30,31,32,33,34,35,36,37,38,39,40,41,42,43,44,45] were included in the systematic review after the removal of duplicate articles and the exclusion of irrelevant studies through perusal of the titles, abstracts, and full texts (Figure 1).

### 3.1. Study Characteristics

The recruited participants comprised a total of 375 healthy adult subjects (226 males and 149 females, age range: 18–40 years). All twenty studies [18,19,20,28,29,30,31,32,33,34,35,36,37,38,39,40,41,42,43,44] employed randomized crossover experimental designs, except for one study [45], which was a randomized parallel trial. The participants visited the laboratory on multiple occasions and were randomized to receive single anodal tDCS and sham stimulation (Sham), which involved the brief delivery of a current of the same intensity. This method enabled the comparison of the immediate effects of various stimulation conditions on lower-limb endurance performance. The included studies can be categorized based on the involvement of the overall lower limb (Table 1) or the focus on a single joint of the lower limb (Table 2), depending on the fatigue protocol. The major outcomes of the studies included the following: (1) Fatigue index: quantified via the percentage decline in torque production from beginning to end of the fatigue protocol [39,41,43,44]; (2) TTF: calculated as the difference between the time at task failure and start time [18,19,20,28,29,32,33,34,35,36]; (3) Rate of perceived exertion (RPE): measured using the Borg scale [18,19,28,29,30,36,37] or a visual analogue scale [33].

### 3.2. Effects of tDCS on Endurance Performance of Overall Lower Limbs

**Figure 2:**
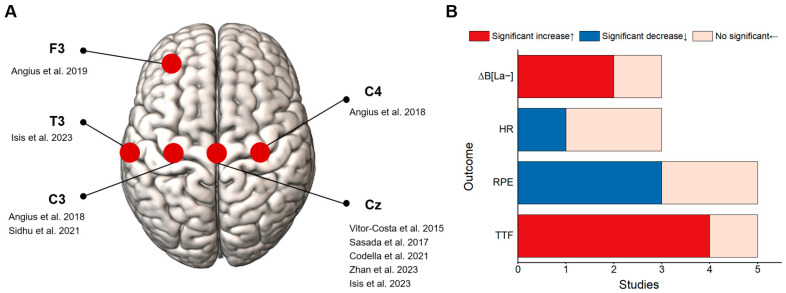
Characteristics of studies involving the overall lower limbs [18,19,20,28,29,30,31,45]. (**A**) List of studies that targeted different brain regions of interest. (**B**) Summary of study results obtained using key outcome indicators.

Eight studies were included [18,19,20,28,29,30,31,45], each with significant variations in the tDCS protocols used. The primary stimulated brain area was the primary motor cortex (M1) in seven of the eight studies (87.5%), with only one study targeting the left dorsolateral prefrontal cortex (DLPFC) (Figure 2A). Although the seven studies targeted M1 for stimulation [18,19,20,29,30,31,45], the effect on endurance performance varied, with only three studies revealing remarkable improvement in TTF [18,19,29]. Another work that focused on F3 reported a significant enhancement in TTF [28], which suggests that M1 may not be the only effective target for the improvement of endurance performance. Except for one study that used stimulation headphones and another that employed 4 × 1 high-definition (HD) tDCS, six studies utilized large electrode pads with sizes ranging from 25 cm^2^ to 36 cm^2^ and applied a 2 mA current intensity for 10–30 min of stimulation. All studies used an offline stimulation mode and administered stimulation prior to the implementation of the fatigue protocol (Table 1).

Four studies used a cycling fatigue protocol of 70% to 80% of the maximal power output; the anodal tDCS considerably prolonged cycling TTF [18,19,28,29], increased blood lactate accumulation (∆B[La−]), and reduced the RPE during cycling [19,28] (Figure 2B). Among the four studies that reported a notable increase in TTF, one used a stimulation duration of 30 min, and the other three used durations shorter than 15 min. This finding implies that the stimulation duration may not be a major factor influencing the effectiveness of the intervention. Two studies that used a running-induced fatigue protocol revealed that tDCS extensively elevated maximal oxygen uptake and muscle activity in the dorsal flexors and increased cortical muscle coherence [30,45]. However, another study revealed that tDCS did not substantially enhance the 30 s sprint cycling power performance [31]. Furthermore, two studies reported that tDCS increased the lower-limb corticospinal excitability [19,20]. Overall, the articles that reported notable improvements in the endurance performance almost always used large electrodes, with sizes ranging from 25 cm² to 36 cm². Although large electrodes may modulate a wide cortical area, which leads to a pronounced influence, they may also cause unintended side effects. Conversely, small electrodes can target specific areas precisely but may result in diminished effects.

### 3.3. Effects of tDCS on Endurance Performance of a Single Joints/Segments of Lower Limbs

**Figure 3:**
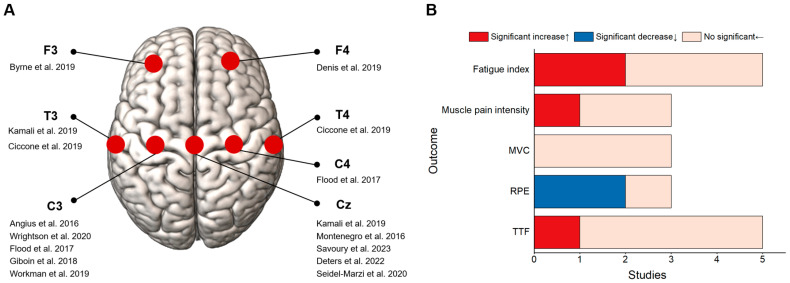
Characteristics of studies involving single joints/segments of lower limbs [32,33,34,35,36,37,38,39,40,41,42,43,44]. (**A**) List of studies that targeted different brain regions of interest. (**B**) Summary of study results obtained using key outcome indicators.

In this section, thirteen studies were included [32,33,34,35,36,37,38,39,40,41,42,43,44]; aside from three studies, i.e., two that stimulated the prefrontal cortex [33,35] and one that targeted the temporal lobe [39], the M1 area was the primary stimulated brain region (10 studies, 76.9%) (Figure 3A). In addition to the two studies that employed 4 × 1 high-definition tDCS, 11 studies utilized large electrode pads with sizes ranging from 12 cm^2^ to 100 cm^2^. The electrical current intensity was set at 2 mA in eleven studies, and stimulation lasted from 10 min to 30 min. Four studies employed an online stimulation mode, which involved the concurrent application of the electrical stimulation with fatigue (Table 2).

Four studies revealed the failure of tDCS to considerably increase the TTF during 20–30% maximal voluntary contractions (MVC) isometric contractions of the knee [32,33,34,35]. Two of these studies revealed no extensive improvement in muscle pain intensity or fatigue perception [32,35] (Figure 3B). However, two studies demonstrated that tDCS greatly enhanced the TTF of isometric contraction and short-term endurance indices of the knee, and both significantly reduced RPE [36,37]. Five studies used a rapid fatigue protocol for a single joint in the lower limbs and revealed that none of the tDCS failed to greatly improve the total work or reduce the fatigue index [38,39,40,41,43]. A previous work that focused on female participants and used a 4 mA current intensity reported that tDCS considerably increased the lower-limb muscle activation levels and fatigue index during the high-estrogen phase [44]. Furthermore, one study that focused on the lower-limb ankle joint reported that tDCS enhanced the capability to sustain rapid tapping frequencies [42].

### 3.4. Risk of Bias Assessment

**Figure 4:**
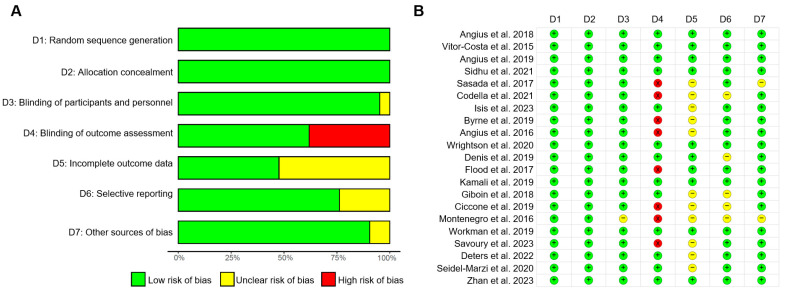
Risk of bias assessment [18,19,20,28,29,30,31,32,33,34,35,36,37,38,39,40,41,42,43,44,45]. (**A**) Risks of bias were represented by percentages. (**B**) Risks of bias summary. + low risk of bias; − high risk of bias; × unclear risk of bias.

The included studies showed varying levels of the risk of bias (Figure 4). All twenty-one studies employed random grouping. In eight studies, blinding was applied only to the participants. Ten studies unveiled the completeness of outcome measures, and the remaining ones did not provide such information. The risk of selective reporting bias was unclear in five articles, and the sources of other biases were unclear in two.

## 4. Discussion

The present literature review reveals that tDCS considerably enhances the endurance performance indices, such as TTF, peak oxygen uptake, etc., of the overall lower limb compared with the sham stimulus. However, this process may not necessarily improve performance indicators, such as TTF, work output, and fatigue index, in knee endurance exercise. This discrepancy can be attributed to the complexity of factors affecting performance in different endurance exercises and various mechanisms underlying the effects of tDCS on endurance performance.

### 4.1. Some Potential tDCS Mechanisms That Have Been Suggested to Improve Lower-Limb Endurance Performance

In studies where tDCS remarkably enhanced lower-limb endurance performance compared with the control group, post-tDCS intervention exhibited a lower RPE [19,28,37]. The reduction in RPE shows a close association with improved endurance performance. During endurance exercise, RPE reflects an individual’s subjective perception of effort and fatigue level. Currently, the most widely recognized potential mechanism by which tDCS improves endurance performance is that tDCS increases the excitability of M1, which reduces the reliance on the SMA and decreases an individual’s perception of fatigue during exercise [15,46]. Studies have shown that the SMA, as an upstream region of the M1, bears a close relation to the generation of fatigue perception [47]. The SMA also participates in motor initiation, planning, and execution, particularly in complex tasks or those requiring sustained efforts [48]. Previous work has demonstrated that as SMA activation increases, especially when the task becomes challenging or fatigue accumulates, the brain’s perception of effort also intensifies, which leads to an increase in RPE [15]. Functional imaging and electrophysiological studies have demonstrated that SMA activity is closely linked to the perception of fatigue and motor output [49,50]. During exercise fatigue, SMA activity increased with increased muscle activity, leading to increased fatigue and decreased motor output [46]. tDCS can increase the excitability of the M1 region through the alteration of the resting membrane potential of neurons [51], which increases the susceptibility of the M1 region to commands from other areas [52]. Therefore, compared with the control group, during an exercise of the same intensity, the SMA required a lower level of activation to transmit the corresponding information commands to the M1 region [19,28], which reduced the perception of fatigue (i.e., lowering RPE) during lower-limb endurance exercise. These alterations motivate individuals to sustain target force output durably during endurance exercise, which enhances the TTF in endurance exercise [19,28]. Some studies speculate that tDCS may reduce muscle pain to improve endurance, but this inference has not been fully substantiated [53]. Moreover, based on the importance of the DLPFC for inhibitory control and motor regulation, studies have also attempted to improve endurance performance by modulating the DLPFC. Studies have shown that anodal tDCS of the left DLPFC improves overall lower-limb endurance performance while improving inhibitory control and reducing perceived effort [28]. However, two other studies using anodal tDCS of the DLPFC did not show improvements in single-joint endurance performance [33,35]. Therefore, further empirical studies are required to confirm the effect of anodal tDCS on DLPFC on the improvement of endurance performance.

### 4.2. Possible Reasons for the Inconsistent Effects of tDCS Intervention

The tDCS exhibited a notably better intervention effect on overall lower-limb endurance exercises than on knee endurance exercises. This difference is attributed to variations in fatigue mechanisms between the two types of endurance exercises [54]. During overall endurance exercises, individuals typically cease exercising due to central fatigue (i.e., the inability of the central nervous system to adequately drive motoneurons) [15,55]. Therefore, tDCS can increase the endurance performance by boosting the excitability of the M1 region. However, during single-joint exercises, such as knee endurance exercises, individuals experience less central fatigue but more peripheral fatigue [56]. Peripheral fatigue is caused by changes distal to the neuromuscular junction, which impairs muscle contraction and ultimately leads to cessation of movement [55,57]. Therefore, the direct effect of tDCS on single-joint endurance performance may not be significant. In addition, the majority of whole-limb and single-joint studies have used large electrodes. Previous studies have demonstrated that the use of larger sponge electrodes produces diffuse rather than focused currents, resulting in a lack of focality [58,59]. The use of larger sponge electrodes may be more effective in improving overall endurance performance, but in a single-joint task, these electrodes are unable to precisely modulate brain regions associated with a particular task [60,61], resulting in smaller improvements in single-joint endurance performance [34,35].

In addition, within knee endurance exercises, discrepancies were detected in the reported effects on endurance performance across various studies. Several studies have shown that tDCS failed to enhance cortical excitability [32,38], possibly due to the various tDCS stimulation protocols used. The tDCS that was applied only to the temporal cortex failed to improve knee endurance performance [31], whereas simultaneous multitarget stimulation of M1 and temporal cortex considerably improved knee endurance performance [37]. Furthermore, the tDCS that targeted the primary motor cortex had various intervention effects on the endurance performance of knee joint isometric contractions [32,36]. This variability may stem from differences in the size of the electrodes used in the two experiments, which led to variations in the coverage of brain regions. The use of electrode pads of different sizes resulted in various ranges of brain areas covered. The conventional tDCS with large electrodes may induce a broad electric field distribution in the target brain area, which can interfere with the researchers’ ability to acquire accurate research results and mechanistic explanations [34]. By contrast, HD-tDCS, when applied with the same current intensity to cover the same brain region, can increase the focus of stimulation by 80% and the intensity of stimulation at the target point by 98%. This finding allows for the determination of the relationship between the target brain area and changes in performance, which facilitates the exploration of the potential neurophysiological mechanisms underlying the improvement in the endurance performance [62]. In addition, anodal tDCS is believed to depolarize neurons and increase firing rates, whereas cathodal tDCS hyperpolarizes neurons and decreases firing rates [51]. However, this notion is an oversimplification. Neurons cause no uniform depolarization or hyperpolarization in response to the applied current. Conversely, various compartments of the same neuron may depolarize or hyperpolarize simultaneously [63]. Anodal tDCS can also decrease [64], and cathodal tDCS can increase corticospinal excitability [65]. Overall, tDCS exerts an inconsistent intervention effect on knee endurance performance. Future research should not only investigate whether a single tDCS protocol can improve endurance performance, but also compare the effects of different tDCS protocols, which would help identify the optimal protocol for improving endurance performance.

### 4.3. Limitations

The optimal montage of tDCS (e.g., appropriate cortical target, current intensity, and duration) for the improvement of endurance performance remains challenging given the high variability of tDCS protocols in current publications. In addition, although studies have detected some improvements in the overall lower-limb endurance performance with tDCS, the mechanisms by which tDCS may enhance physical function remain largely unclear. The effects of anodal and cathodal tDCS on cortical activation and functional performance must be investigated to gain insights into the causal role of brain activity in the regulation of endurance activities. Finally, the current research failed to explore the neurophysiological mechanisms underlying the effects of tDCS on endurance performance. The application of neuroimaging techniques, such as functional magnetic resonance imaging [66], functional near-infrared spectroscopy [67], and other related methods, may contribute to the exploration of the mechanisms of tDCS modulation in endurance performance. Nonetheless, the findings of this work offer important knowledge on the effects of tDCS on endurance performance.

## 5. Conclusions

tDCS, which is a noninvasive neuromodulation technique, showed a crucial effect on overall lower-limb endurance performance. tDCS can increase the excitability of the primary motor cortex, which reduces the activation of the SMA and lowers the perception of fatigue during endurance exercise. Future research may focus on the application effects of multitarget stimulation, personalized dosages, and other stimulation protocols. The development targeted stimulation protocols can improve antifatigue capabilities based on different needs.

## Figures and Tables

**Table 1:** Effect of tDCS on endurance performance of overall lower limbs.

Study	Sample (Male/Female)	Age (Years)	tDCS Protocol				Fatigue Protocol	Main Outcomes
			Anodal/Cathodal Location	Electrode Size (cm^2^)	Current (mA)	Duration (min)		
Angius et al, 2018 [19]	8/4	24 ± 5	A: bilateral M1, C: ipsilateral shoulders/A: ipsilateral shoulders, C: bilateral M1	A: 35, C: 25 A: 25, C: 35	2	10	70% W_peak_ Cycling	TTF↑ corticospinal excitability (VL)↑ ∆B[La−]↑ RPE↓
Vitor-Costa et al, 2015 [18]	11/0	26 ± 4	A: Cz, R: occipital protuberance/C: Cz, R: occipital protuberance	A: 36, C: 35	2	13	80% W_peak_ Cycling	TTF↑ peak power← RPE← HR← sEMG activity (VL)←
Angius et al, 2019 [28]	9/3	23 ± 3	A: F3, C: Fp2	A: 35, C: 25	2	30	70% W_peak_ Cycling	TTF↑ RPE↓ ∆B[La−]↑ HR↓
Sidhu et al, 2021 [29]	12/0	20.8 ± 0.4	A: left M1, C: right supraorbital	25	2	10	80% W_peak_ Cycling	TTF↑ RPE← HR←
Sasada et al, 2017 [31]	17/6	21~30	A: vertex, C: right forehead/A: right forehead, C: vertex	35	2	15	30s maximum effort sprint cycling	pooled mean power← peak power←
Codella et al, 2021 [30]	17/0	30.9 ± 6.5	portable tDCS headset: Cz, C1–C6	3 × 28	2	20	modified Bruce ramp protocol	VO_2peak_↑ RPE↓
Zhan et al, 2023 [45]	24/0	A: 21.5 ± 2.2 C: 21.7 ± 2.3	A: Cz, C:C3, C4, Fz, Pz	4 × 1 HD-tDCS	2	20	running-induced fatigue	sEMG activity (TA)↑ CMC (beta: C1-TA)↑ CMC (gamma: C1-TA, Cz-TA)↑
Isis et al, 2023 [20]	6/9	25.8 ± 5	A: MI, C: T3/A: T3, C: M1	35	2	20	maximal incremental exercise test (cycling)	TTF← sEMG activity (VL, RF, VM)← cortical excitability (VL)↑

Note: A = anodal; C = cathodal; R = reference electrode; M1 = primary motor cortex; Cz, C1, C3, C4, C6, F3, Fp2, and T3 are international EEG 10–20 system electrode placement sites; W_peak_ = peak power output; TTF = time to task failure; VL = vastus lateralis; ∆B[La−] = blood lactate accumulation; RPE = ratings of perceived exertion; HR = heart rate; sEMG = surface electromyography; VO_2peak_ = peak oxygen uptake; HD-tDCS = high-definition tDCS; TA = tibialis anterior; CMC = corticomuscular coherence; RF = rectus femoris; VM = vastus medialis; compared with the sham stimulation group, after the tDCS intervention: ↑ significant increase; ↓ significant decrease; ← no significant changes.

**Table 2:** Effect of tDCS on the endurance performance of a single joint/segment of lower limbs.

Study	Sample (Male/Female)	Age (Years)	tDCS Protocol				Fatigue Protocol	Main Outcomes
			Anodal/Cathodal Location	Electrode Size (cm^2^)	Current (mA)	Duration (min)		
Byrne et al, 2019 [35]	11/12	26 ± 5	A: F3, C: Fp2	35	2	20	25% MVC isometric KE	TTF← MVC← muscle pain intensity←
Angius et al, 2016 [36]	9/0	23 ± 2	A: left M1, C: right prefrontal cortex/A: left M1, C: left shoulder	12	2	10	20% MVC isometric KE	TTF↑ RPE↓ muscle pain intensity←
Wrightson et al, 2020 [32]	11/9	23.8 ± 4.7	A: hotspot for the right VL, C: left deltoid	35	1, 2	10	20% MVC isometric KE	TTF← sEMG activity (VL)← cortical excitability (VL)← perceived fatigue← MVC←
Denis et al, 2019 [33]	7/13	20.6 ± 1.7	A: right dorsolateral prefrontal cortex, C: distance of 3.5 cm around the anode electrode	4 × 1 HD-tDCS	2	10+ (online)	30% MVC isometric KE	TTF← RPE←
Flood et al, 2017 [34]	12/0	24.4 ± 3.9	A: C3, C: Cz, F3, T7, P3/A: C4, C: Cz, F4, T8, P4	4 × 1 HD-tDCS	2	20	30% MVC isometric KE	TTF← endogenous pain inhibition↑ MVC←
Kamali et al, 2019 [37]	12/0	18~40	A: M1 + T3, C: bilateral shoulder	A: 35, C:16/A:16, C: 16	2	13	30% 1 RM isotonic KE	SEI↑ RPE↓ HR↓ 1 RM↑ sEMG activity (RF)↑
Giboin et al, 2018 [38]	14/0	26 ± 3	A: hotspot for the right VL, C: contra lateral orbit	35	2	10 (online)	35 × 5 s MVC isometric KE	amplitude of MVC↓ sEMG activity (VL)↓
Ciccone et al, 2019 [39]	10/10	21.0 ± 1.5	A: T3, C: Fp2 /A: T4, C: Fp1	25	2	30	50 maximal effort isokinetic KE	fatigue index← mean torque integral← HR variability←
Montenegro et al, 2016 [40]	13/0	26 ± 4	A: M1, C: Fp2	35	2	20	3 × 10 maximal effort isokinetic KE	total work← work fatigue percentage← peak torque← sEMG activity (VM, RF, BF, ST)←
Workman et al, 2019 [41]	12/22	24 ±3.6	A: C3, C: contralateral supraorbital area	35	4	20 (online)	40 maximal effort isokinetic KE and KF	fatigue index (KF muscle group)↑ fatigue index (KE muscle group)←
Savoury et al, 2023 [43]	8/8	males 24.1 ± 2.8 females 21.9 ± 1.6	A: M1, C: ipsilateral shoulder area	A: 25; C: 35	2	10	12 × 5 s maximal effort isokinetic KE	MVC (KE)↓ normalized MVC (KE)↓ fatigue index←
Deters et al, 2022 [44]	0/10	24.3 ± 5.5	A: M1, C: Fp2	A: 25; C: 35	4	20	40 120°/s maximal effort isokinetic KE and KF	fatigue index (during high-estrogen level)↑ sEMG activity (KE and KF)↑
Seidel-Marzi et al, 2020 [42]	FB: 10/3 HB: 7/5 NA: 10/11	FB: 24.0 ± 3.9 HB: 22.5 ± 4.3 NA: 27.0 ± 3.4	A: Cz, C: Fz	A: 35; C: 100	2	20 (online)	20 s foot-tapping tasks	maintenance of tapping frequency↑

Note: A = anodal; C = cathodal; M1 = primary motor cortex; Cz, C3, C4, T3, T4, T7, T8, F3, F4, P3, P4, Fp1, and Fp2 are international EEG 10–20 system electrode placement sites; MVC = maximal voluntary contractions; KE = knee extensors; KF = knee flexion; TTF = time to task failure; RPE = ratings of perceived exertion; sEMG = surface electromyography; VL = vastus lateralis; HD-tDCS = high-definition tDCS; SEI = short-term endurance index; HR = heart rate; 1RM = one-repetition maximum; RF = rectus femoris; VM = vastus medialis; BF = biceps femoris; ST = semitendinosus; FB = football player; HB = handball player; NA = nonathletes; compared with the sham stimulation group, after the tDCS intervention: ↑ significant increase; ↓ significant decrease; ← no significant changes.

## Data Availability

The data are included in the article, further inquiries can be directed to the corresponding author/s.
